# Sulfotransferase SULT2B1 contributes to the epithelial–immune microenvironment homeostasis in imiquimod-induced psoriatic dermatitis

**DOI:** 10.3389/fimmu.2025.1632426

**Published:** 2025-10-17

**Authors:** Kenji Morino, Sayaka Akiyoshi, Keisuke Matsubara, Yuki Sugiura, Yoshihiro Izumi, Shu Yotsumoto, Kazuhiko Yamamura, Rae Maeda, Masatomo Takahashi, Keisuke Nakata, Takeshi Bamba, Takeshi Nakahara, Daiji Sakata, Takehito Uruno, Yoshinori Fukui, Kazufumi Kunimura

**Affiliations:** ^1^ Division of Immunogenetics, Department of Immunobiology and Neuroscience, Medical Institute of Bioregulation, Kyushu University, Fukuoka, Japan; ^2^ Multiomics Platform, Center for Cancer Immunotherapy and Immunobiology, Graduate School of Medicine, Kyoto University, Kyoto, Japan; ^3^ Division of Metabolomics, Medical Research Center for High Depth Omics, Medical Institute of Bioregulation, Kyushu University, Fukuoka, Japan; ^4^ Department of Dermatology, Graduate School of Medical Sciences, Kyushu University, Fukuoka, Japan; ^5^ Department of Immunology, Graduate School of Medical and Dental Sciences, Kagoshima University, Kagoshima, Japan

**Keywords:** SULT2B1, cholesterol sulfate, DOCK2, skin inflammation, psoriasis, neutrophils

## Abstract

**Introduction:**

Skin protects the body from external threats by constituting an epithelial–immune microenvironment. Sulfotransferase family 2B member 1 (SULT2B1) converts cholesterol to cholesterol sulfate (CS). We previously reported that CS acts as an endogenous dedicator of cytokinesis 2 (DOCK2)-inhibitory metabolite suppressing immune cell migration and activation by inhibiting DOCK2-mediated Rac activation. Despite being located in the epidermis, pathophysiological roles of CS in cutaneous inflammation remain unknown.

**Methods:**

We evaluated the *Sult2b1*-producing cells in the dorsal skin of wild-type mice and compared the degree of cutaneous inflammation between wild-type and *Sult2b1* knockout mice using a psoriatic dermatitis model induced by topical imiquimod (IMQ). We also examined *SULT2B1* gene expression levels in human epidermal keratinocytes to assess the effects of pro-inflammatory cytokines.

**Results:**

*Sult2b1* expression levels and CS production gradually increased in the skin of psoriatic dermatitis model mice. IMQ-induced dermatitis and neutrophil recruitment were exacerbated in the *Sult2b1* knockout mice with a complete loss of CS. Furthermore, genetic deletion of *Dock2* or intravenous administration of neutrophil-depleting antibodies alleviated IMQ-induced dermatitis in *Sult2b1* knockout mice. Notably, CS was more abundant in the skin samples of patients with psoriasis than in the healthy control samples. Primary normal human epidermal keratinocytes exhibited significantly elevated *SULT2B1* levels after Th1 cytokine treatment.

**Discussion:**

These findings suggest that increased SULT2B1 levels in the skin under psoriatic conditions may be involved in a negative feedback mechanism that helps to limit excessive skin inflammation, thereby potentially contributing to the maintenance of epithelial–immune microenvironment homeostasis. Overall, our results raise the possibility that SULT2B1 plays an important role in cutaneous inflammation and could serve as a useful indicator or potential target in psoriasis.

## 1 Introduction

Skin plays protective roles in the human body by acting as a physical and immunological barrier against chemical hazards and pathogens threatening homeostasis. To defend against external threats, cutaneous immune responses primarily occur in the epidermis and papillary dermis in superficial skin sites, which constitute the epithelial–immune microenvironment (EIME) (1). Cytokines produced by immune cells in inflamed skin influence the activation and proliferation of non-immune cells, such as keratinocytes (KCs). These cytokines form a complex inflammatory network in EIME, often leading to inflammatory skin diseases (2, 3). Psoriasis is a chronic inflammatory skin disease characterized by erythema, scaling, and skin thickening attributed to an overactive immune system (4). Genetic predisposition is associated with pro-inflammatory KC signaling and type 17 immune responses. Psoriasis typically involves a feed-forward loop, which amplifies local inflammation and possibly also influences its exacerbation and remission (5, 6). However, specific inflammation regulatory mechanisms remain unknown.

Dedicator of cytokinesis 2 (DOCK2) is a mammalian homolog of *Caenorhabditis elegans* CED-5 and *Drosophila melanogaster* myoblast city (7). It is predominantly expressed in the hematopoietic cells and functions as a critical Rac guanine nucleotide exchange factor (GEF) downstream of chemoattractant receptors and antigen receptors in immune cells (8–10). It also plays a fundamental role in immune surveillance in mice and humans by positively regulating immune cell migration and activation (11–13). We previously explored potential molecules impeding the Rac GEF activity of DOCK2 and identified cholesterol sulfate (CS) as an endogenous DOCK2-inhibitory metabolite (14). CS is a sulfated derivative of cholesterol mediated by SULT2B1, a member of the sulfotransferase (SULT) family of sulfate-conjugating enzymes (15). It directly binds to the catalytic domain of DOCK2, thereby suppressing immune cell migration and activation (14). In SULT2B1-deficient (*Sult2b1^−/−^*) mice, CS production was abolished in the CS-producing tissues, suggesting the critical role of SULT2B1 in CS production (14). SULT2B1 is produced in the epidermis and involved in epidermal cell differentiation (16); however, its physiological roles in EIME remain ambiguous.

In this study, we evaluated the *Sult2b1*-producing cells in the dorsal skin of *Sult2b1^+/+^* mice and compared the degree of cutaneous inflammation between *Sult2b1^+/+^* and *Sult2b1^−/−^* mice using an imiquimod (IMQ; toll-like receptor 7 agonist)-induced psoriatic skin inflammation model to determine the specific roles of SULT2B1 in EIME under inflammatory conditions. Additionally, we assessed the most variable immune cell types in the skin of IMQ-treated *Sult2b1^−/−^* mice and explored the impact of the CS–DOCK2 axis. We also used human epidermal KCs to determine the effects of pro-inflammatory cytokines mainly produced by T helper (Th)-1, Th2, and Th17 cells on *SULT2B1* mRNA expression levels. Our data suggest that SULT2B1, expressed in epidermal KCs, plays a key role in maintaining EIME homeostasis by partially suppressing the excessive immune cell responses.

## 2 Materials and methods

### 2.1 Mice

*Sult2b1-tdTomato* knock-in mice were established as previously described (17). *Sult2b1* ^−/−^ mice were obtained from the Jackson Laboratory (stock no. 018773; Bar Harbor, ME, USA). *Dock2* ^−/−^ mice have been previously established (8). *Sult2b1* ^−/−^ and *Dock2* ^−/−^ mice were backcrossed with C57BL/6J mice for over 10 generations before use. C57BL/6J mice were purchased from CLEA Japan (Tokyo, Japan). All mice were housed under light-controlled conditions (12/12-h light/dark cycle), with free access to food and water. Age-matched male *Sult2b1* ^+/+^ and *Sult2b1* ^−/−^ littermates were used at 8–12 weeks of age. The mice were randomly divided into experimental groups according to their genotypes. The sample size for each group was determined according to previous reports using similar experimental models (14, 17–21), and no statistical methods were used to predetermine sample sizes. The number of animals used in this study is indicated in the captions for each figure. All mice were maintained under specific pathogen-free conditions at the animal facility of Kyushu University (Fukuoka, Japan). All animal experiments were conducted according to the relevant national and international guidelines described in the Act on Welfare and Management of Animals (Ministry of Environment of Japan) and Regulation of Laboratory Animals (Kyushu University) guidelines. All animal experiments were approved by the Ethics Committee on Animal Experiments of Kyushu University (approval numbers: A22-032, A22-142, A24-107, and A24-199).

### 2.2 Establishment of an IMQ-Induced skin inflammation model

To induce psoriatic skin inflammation, the mice were topically treated with IMQ, as previously described (18). Shaved dorsal skin was treated with 32 mg IMQ cream (5% IMQ; Mochida Pharmaceutical, Tokyo, Japan) daily for five consecutive days, translating to a daily dose of 1.6 mg of the active compound. Skin inflammation severity was evaluated using the clinical scores based on the Psoriasis Area Severity Index (19). Erythema, scaling, and thickening were scored on a scale from 0 to 4 (0 = none, 1 = slight, 2 = moderate, 3 = marked, and 4 = severe), and addition of these three parameters yielded a cumulative score in the range of 0–12, which was further used to measure the inflammation severity. Trans epidermal water loss (TEWL) was measured using VAPO SCAN (AS-VT100RS; ASCH JAPAN Co., Ltd., Tokyo, Japan), according to the manufacturer’s instructions.

### 2.3 CS quantification and imaging via mass spectrometry

The mice were decapitated following isoflurane anesthesia, and skin tissues were dissected from the shaved dorsal skin, snap-frozen in liquid nitrogen, and stored at −80°C until analysis. CS levels in skin samples were quantified via MS, as previously described (20). Briefly, frozen samples were homogenized and mixed with 1 mL of methanol, including 147.5 pmol of internal standard (deuterium-labeled CS; d7-CS; #903752; Sigma-Aldrich, St Louis, MO, USA). The extracts were then centrifuged, and the resulting supernatant (410 μL) was mixed with 410 μL of chloroform and 328 μL of water. Phase separation of the aqueous and organic layers was performed by centrifugation. The organic (lower) layer (280 μL) was placed in another tube, dried with nitrogen gas, and stored at −80°C. The reconstituted sample, diluted to 40 μL with methanol, was analyzed by liquid chromatography-MS/MS to determine the CS levels. Triple-quadrupole MS equipped with an electrospray ionization ion source (LCMS-8060; Shimadzu Corporation, Kyoto, Japan) was performed in negative electrospray ionization and multiple reaction monitoring (MRM) modes. CS and d7-CS signals were monitored as ion transitions at mass/charge ratio (*m/z*) 465.3 > 96.9 and 472.3 > 96.9, respectively. Absolute CS content was calculated based on the peak area ratio of CS to the internal standard, d7-CS. The linear dynamic range of CS was evaluated using commercially available CS of known concentration (#15106; Cayman Chemical, Ann Arbor, MI, USA) and its dilution series, through three technical replicates (**Supplementary Figure S1**).

Data were processed using Multi-ChromatoAnalysT v.1.3.4.0 (BeForce Co., Fukuoka, Japan). The details of the analytical conditions for the analyses of CS were as follows: injection volume, 5 μL; column temperature, 50°C; mobile phase A, 5 mM ammonium acetate in water/acetonitrile (1/2, v/v); mobile phase B, 5 mM ammonium acetate in methanol/isopropanol (1/19, v/v); and flow rate, 0.3 mL/min. The gradient conditions were as follows: 0−100% B, 0−22 min; 100% B, 22−27 min; and 0% B, 27.1−35 min. The MRM analysis conditions were as follows: polarity, negative ionization; electrospray voltage, –3 kV; nebulizer gas flow rate, 3 L/min; drying gas flow rate, 10 L/min; desolvation line temperature, 250°C; heat block temperature, 400°C; and detector voltage, 2.44 kV.

For CS imaging, matrix-assisted laser desorption/ionization (MALDI) imaging analysis was performed as previously described (21). Briefly, thin skin tissue sections (8 μm) were prepared using a cryomicrotome (CM3050; Leica Microsystems, Wetzlar, Germany) and supported using the Kawamoto cryofilm (SECTION-LAB Co. Ltd., Kanagawa, Japan). The tissue sections were mounted on indium tin oxide-coated glass slides (Bruker Daltonics, Bremen, Germany). MALDI-linear ion trap MS (MALDI LTQ XL; Thermo Fisher Scientific, Waltham, MA, USA) and ultrafleXtreme MALDI-TOF/TOF (Bruker Daltonics) were used for MALDI imaging analysis of CS. Data were acquired on the TOF/TOF and LTQ instruments in the negative reflectron and selected ion monitoring modes, respectively, with raster scans at a pitch distance of 30 μm. Image reconstruction of TOF/TOF data was performed using the FlexImaging 4.1 software (Bruker Daltonics), and LTQ data were visualized using the ImageQuest v.1.0.1 software (Thermo Fisher Scientific).

### 2.4 Western blotting

Skin tissues were homogenized using a 2× cell lysis buffer (#9803; CST, Danvers, MS, USA) supplemented with complete protease inhibitors (Roche, Basel, Switzerland) using an electric homogenizer for 1 min on ice. After centrifugation, the supernatant was mixed with an equal volume of 2× sample buffer (125 mM Tris-HCl, 0.01% bromophenol blue, 4% sodium dodecyl sulfate, 20% glycerol, and 200 μM dithiothreitol) and boiled for 10 min. Total protein concentration was measured using the DC Protein Assay Reagent (Bio-Rad, Hercules, CA, USA). Tissue extracts were separated via sodium dodecyl-sulfate polyacrylamide gel electrophoresis (FUJIFILM Wako, Osaka, Japan) and immunoblotted with the rabbit anti-SULT2B1b (custom-made (14); 1:1,000) and goat anti-β-actin (#sc-1616; 1:2,000; Santa Cruz Biotechnology, Dallas, TX, USA) antibodies. The following horseradish peroxidase-conjugated secondary antibodies were used: Mouse anti-rabbit IgG (#sc-2357; 1:2,000; Santa Cruz Biotechnology) and mouse anti-goat IgG (#sc-2354; 1:2,000; Santa Cruz Biotechnology).

### 2.5 Single-cell RNA-sequencing and MS data analyses

Gene expression in each mouse dorsal skin subset was analyzed using previously reported single-cell RNA-seq data (22). Skin cell samples were collected from four 10−15 weeks old male and four virgin female C57BL/6JN mice. Fluorescence-activated cell sorting (FACS)-based full-length transcript data were used in this study. The processed data, protocols, and analysis scripts from *Tabula Muris* have been shared as public resources (https://tabula-muris.sf.czbiohub.org). The bioinformatics methods and data processing details have been previously described (22). Violin plots and t-distributed stochastic neighbor embedding (t-SNE) visualization for specific gene expression were performed using a publicly available web tool (https://tabula-muris.sf.czbiohub.org/visualizations; provided by the Tabula Muris Consortium).

Epidermal CS levels in the tape-stripped samples of patients with seborrheic keratosis, actinic keratosis, tinea corporis, atopic dermatitis, and psoriasis were analyzed using previously reported lipid-profiling data (23). Patients with psoriasis and atopic dermatitis were not taking systemic immunosuppressive medications and had not received topical medications for seven days before skin tape removal. CS levels were quantified via supercritical fluid chromatography-tandem MS.

### 2.6 Histology and immunofluorescence staining

Skin tissues were fixed with 4% (w/v) paraformaldehyde (FUJIFILM Wako) overnight at 4°C and embedded in paraffin blocks. The sections were stained with hematoxylin and eosin (FUJIFILM Wako) and examined under a light microscope (Axio Lab.A1; Carl Zeiss, Oberkochen, Germany). Skin thickness was quantified using the ImageJ software (v2.9.0/1.53t; National Institutes of Health, Bethesda, MD, USA). For IF staining, tissues were fixed with 4% paraformaldehyde and incubated overnight with 30% sucrose in PBS at 4°C. Then, the samples were embedded in the O.C.T. compound (Sakura Finetech, Tokyo, Japan) and frozen at −80°C. Cryostat (7 μm) was blocked with G-Block (GenoStaff, Tokyo, Japan) for 15 min at room temperature and incubated overnight at 4°C with the following primary antibodies: Rabbit anti-cytokeratin10 (#ab76318; 1:150; Abcam, Cambridge, UK), goat anti-tdTomato (#LS-C340696-600; 1:100; LS Bio, Seattle, WA, USA), rabbit anti-filaggrin (FLG; #905804; 1:100; BioLegend, San Diego, CA, USA), rabbit anti-loricrin (LOR; #905103; 1:100; BioLegend), and biotin-conjugated anti-Gr-1 (#108404; 1:100; BioLegend) overnight at 4°C. Nuclear staining was performed using 4′,6-diamidino-2-phenylindole (Dojindo, Kumamoto, Japan). All images were obtained using a laser-scanning confocal microscope (FV3000; Olympus, Tokyo, Japan).

### 2.7 RNA isolation and real-time polymerase chain reaction analysis

Mouse skin tissues were homogenized in the TRIzol reagent (Thermo Fisher Scientific, Waltham, MA, USA) on ice for 1 min, and total RNA was extracted using the TRIzol Plus RNA Purification Kit (Thermo Fisher Scientific). RNA purity and concentration were assessed using the Nanodrop spectrophotometer (ND-1000; Thermo Fisher Scientific). After treatment with RNase-free DNase I (Thermo Fisher Scientific), RNA samples were reverse-transcribed using Oligo(dT)_12–18_ primers (Thermo Fisher Scientific) and SuperScript III reverse transcriptase (Thermo Fisher Scientific) for PCR amplification. Real-time PCR was performed on the CFX Connect Real-Time PCR Detection System (Bio-Rad) using the SYBR Green PCR Master Mix (Thermo Fisher Scientific). Target gene expression was normalized to that of hypoxanthine phosphoribosyltransferase (*Hprt*). For gene expression analysis, NHEKs were collected, and total RNA was extracted using the RNeasy Mini Kit (#74104; QIAGEN, Valencia, CA, USA). RNA samples were reverse-transcribed using the PrimeScript RT Reagent kit (#RR037; Takara Bio Inc., Kusatsu, Japan). Real-time PCR was performed on the CFX Connect Real-Time PCR Detection System (Bio-Rad) using the TB Green Premix Ex Taq II (RR820; Takara Bio Inc.). Target gene expression was normalized to that of the human acidic ribosomal protein (also known as *RPLP0*), a housekeeping gene for general KC gene expression, as previously described (24, 25). Melting curve analysis was performed to confirm the specificity of the amplification products. All primer sequences are listed in **Supplementary Table S1**. Gene-specific PCR primers for *Lor* were purchased from Bio-Rad (#qMmuCID0008842; PrimePCR SYBR Green Assay; Mouse).

### 2.8 Cytometry by time-of-flight and flow cytometry

Cells were isolated from mouse skin using the Multi Tissue Dissociation Kit 1 (Myltenyi Biotec, Bergisch Gladbach, Germany) and gentleMACS Octo Dissociator with Heaters (Myltenyi Biotec) under the program 37C_Multi_H, according to the manufacturer’s instructions. The homogenized tissues were passed through a 70-μm MACS SmartStrainer (Miltenyi Biotec). Cell staining for CyTOF analysis was performed as previously described (20). The cells were stained with the antibodies listed in **Supplementary Table S2**. Samples were acquired using the Helios CyTOF Mass Cytometer (Standard BioTools, San Francisco, CA, USA). Data were analyzed using the visualization via t-distributed stochastic neighbor embedding algorithm on Cytobank Premium (Cytobank, Inc. Santa Clara, CA, USA).

For flow cytometry analysis, the cells were prepared as described above. Then, the cells were incubated for 10 min at room temperature with the Fixable Viability Stain Reagent (BD Biosciences, Franklin Lakes, NJ, USA), washed, and incubated for 10 min on ice with the anti-mouse CD16/32 antibody (1:1000; 2.4G2; TONBO Biosciences, San Diego, CA, USA). The cells were stained with the following antibodies: Anti-mouse CD45 (1:100; 30-F11; BioLegend, San Diego, CA, USA), anti-mouse CD11b (1:100; M1/70; BD Biosciences), and anti-mouse Gr-1 (1:100, RB6-8C5; BioLegend). Flow cytometry was performed using BD FACSVerse equipped with the BD FACSuite software (BD Biosciences).

### 2.9 *In Vivo* depletion of mouse neutrophils

To examine the effects of neutrophil depletion, anti-neutrophil antibody (NIMP-R14; #AG-20B-0043PF-M002; Adipogen, San Diego, CA, USA) or corresponding isotype control antibody (#AG-35B-0011; Adipogen) were intraperitoneally injected into the mice at a dose of 250 μg before and after IMQ application, as previously described (26). Subsequently, neutrophil depletion in the skin tissues was confirmed via flow cytometry.

### 2.10 Human epidermal cell culture and treatment

Primary normal human epidermal KCs (NHEKs) isolated from adult skin (#CA10205a; Cell Applications, San Diego, CA, USA) were cultured in T-75 flasks with the Human EpiVita Serum-Free Growth Medium Kit for Adult Cells (#A141K500a; Cell Applications) containing 0.06 mM Ca^2+^, according to the manufacturer’s instructions. The medium was refreshed every 2–3 d. The cells with less than four passages were used in all experiments.

NHEKs (1 × 10^5^ cells/well) were plated in a 24-well plate and incubated for 18 h at 37°C and 5% CO_2_. To induce KC differentiation, NHEKs were cultured in a medium containing 1.2 mM Ca^2+^ for 72 h, as previously described (27). Then, the medium was changed, and the cells were treated with each concentration of the cytokines listed in **Supplementary Table S3** for 48 h. The experiment was repeated six times on different days using single-well treatments for each replicate.

### 2.11 Statistical analyses

Statistical analyses were conducted using the Prism 10 software (GraphPad Software, La Jolla, CA, USA). Data were initially tested using the Kolmogorov–Smirnov test for normal distribution. Parametric data were analyzed using a two-tailed unpaired Student’s *t*-test, whereas non-parametric data were analyzed using a two-tailed Mann–Whitney test to compare two groups. Statistical differences among more than two experimental groups were evaluated via one-way analysis of variance, followed by Dunnett’s multiple-comparison test. Data are represented as the mean ± standard deviation. Statistical significance was set at *P* < 0.05.

## 3 Results

### 3.1 Epidermal KCs predominantly express *Sult2b1* in the skin

**Figure 1:**
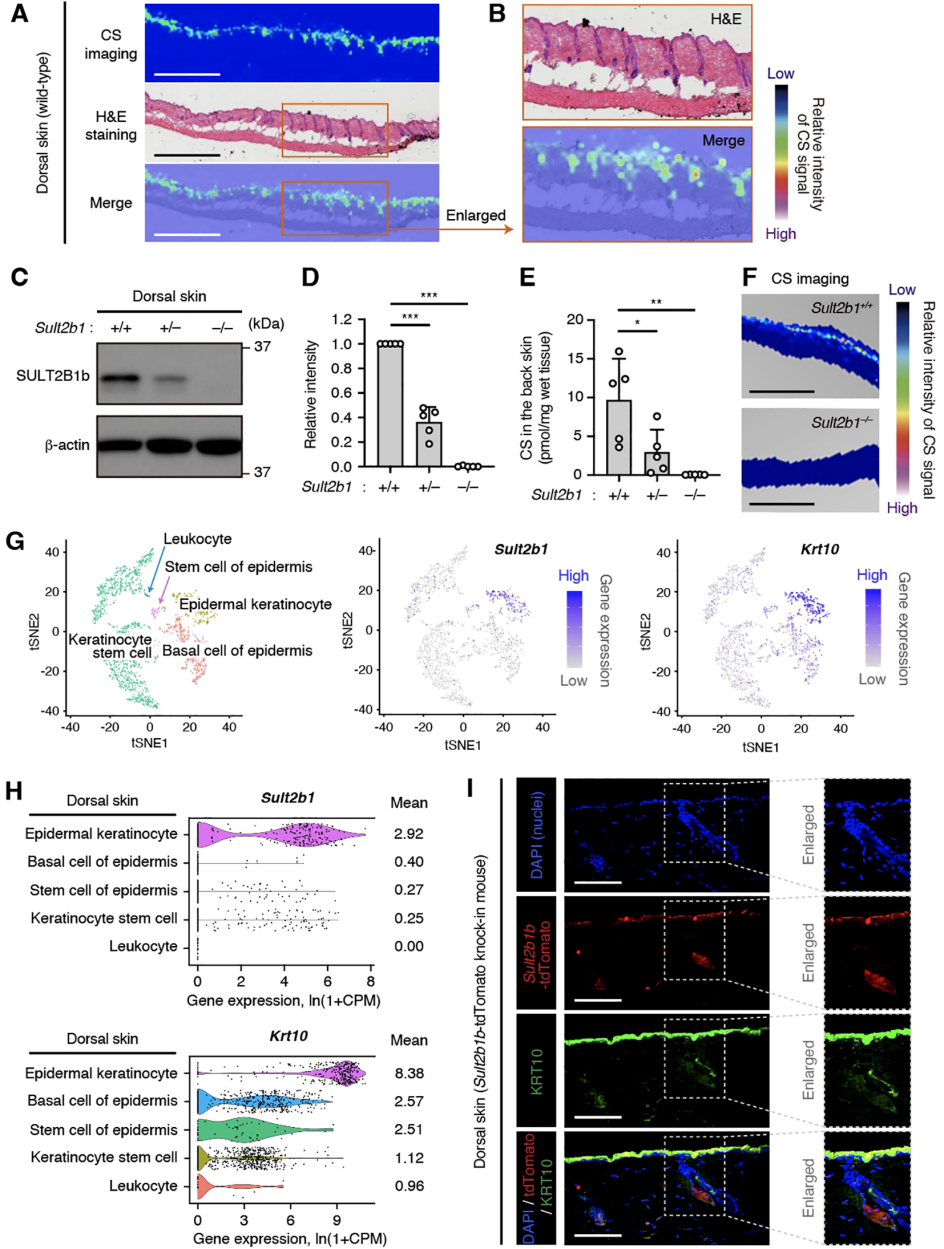
Epidermal keratinocytes predominantly express the sulfotransferase family 2B member 1 (SULT2B1) in the skin. **(A)** Cholesterol sulfate (CS) localization in the dorsal skin sections of *Sult2b1^+/+^* mice at the steady state visualized via mass spectrometry (MS) imaging and hematoxylin and eosin (H&E) staining. Color bar indicates the relative intensity of the CS signal (mass/charge ratio (*m/z*) 465). Scale bar, 1.0 mm. **(B)** Enlarged image of the orange-outlined area in **(A)**. **(C, D)** Representative immunoblots **(C)** showing the SULT2B1b and β-actin levels in the dorsal skin of *Sult2b1^+/+^* mice at the steady state. Bar graphs **(D)** showing the quantification data of five individual blots for each tissue (n = 5; one-way analysis of variance [ANOVA], followed by Dunnett’s multiple-comparison test). **(E)** CS levels in the dorsal skin of *Sult2b1^+/+^*, *Sult2b1^+/−^*, and *Sult2b1^−/−^* mice at the steady state quantified via liquid chromatography-tandem MS (LC-MS/MS; n = 5 mice/group; one-way ANOVA, followed by Dunnett’s multiple-comparison test). **(F)** CS localization in the dorsal skin sections of *Sult2b1* ^+/+^ and *Sult2b1* ^−/−^ mice at the steady state. Color bar indicates the relative intensity of the CS signal (*m/z* 465). Scale bar, 1.0 mm. **(G, H)** t-distributed stochastic neighbor embedding (t-SNE) visualization of mouse dermal cells **(G)**, and violin plots **(H)** showing the gene expression levels of *Sult2b1* and keratin 10 (*Krt10*) in each cell type analyzed using the *Tabula Muris* single-cell RNA-seq data of adult C57BL/6JN mice. Gene count data of the cells sorted via flow cytometry were normalized to counts per million (CPM) and presented as l_n_(1 + CPM). Numbers next to the plots indicate the mean expression levels in each cell type. **(I)** Representative immunofluorescence (IF) staining images showing the tdTomato (red) and KRT10 (green) levels in the dorsal skin of *Sult2b1b-*tdTomato knock-in mice at the steady state counterstained with 4′,6-diamidino-2-phenylindole (DAPI; nucleus; blue). Enlarged (boxed) areas are shown on the right. Scale bar, 100 μm. Data were obtained from two **(A, B)**, five **(C, D)**, two **(E, F),** and three **(I)** independent experiments, and graphs are represented as the mean ± standard deviation (SD). *P < 0.05, **P < 0.01, and ***P < 0.001; ns, not significant.

We previously reported that intestinal epithelial cells at the mucosal–luminal interface in the small and large intestines express SULT2B1 and synthesize CS (20); however, the skin, which is also an interface with the external environment, had not been analyzed in detail. Therefore, in this study, we focused on the dorsal skin of wild-type (WT) mice to visualize CS via MS imaging. CS was produced uniformly on the epidermis, including the hair follicles, of WT mice (**Figures 1A, B**). Next, SULT2B1-deficient mice were used to determine whether SULT2B1 is essential for CS synthesis in the skin. Although SULT2B1 has two isozymes, SULT2B1a and 1b, CS is primarily synthesized by SULT2B1b (14). Therefore, we analyzed the SULT2B1b protein levels in the skin via western blotting and confirmed its absence in *Sult2b1* ^−/−^ mice (**Figures 1C, D**). Its expression levels were also much lower in *Sult2b1* ^+/−^ mice than in *Sult2b1* ^+/+^ mice (**Figures 1C, D**), suggesting that SULT2B1b expression exhibits gene dosage effects. Consistently, CS was absent in the skin of *Sult2b1* ^−/−^ mice, and CS production was significantly decreased in *Sult2b1* ^+/−^ mice (**Figures 1E, F**).

Next, we investigated the specific *Sult2b1*-expressing cell types in the epidermis. Using the single-cell RNA-seq data in public databases, we found that epidermal KCs expressing high levels of keratin 10 (*Krt10*) specifically expressed *Sult2b1* among various mouse dorsal skin subsets (**Figures 1G, H**). IF staining of skin of our previously established *Sult2b1b*-tdTomato knock-in mice (17) also showed that KRT10-positive epidermal KCs expressed *Sult2b1b* (**Figure 1I**). These data suggest that epidermal KCs predominantly express *Sult2b1* and produce CS in the skin.

### 3.2 IMQ-induced skin inflammation upregulates the *Sult2b1* and CS levels

**Figure 2:**
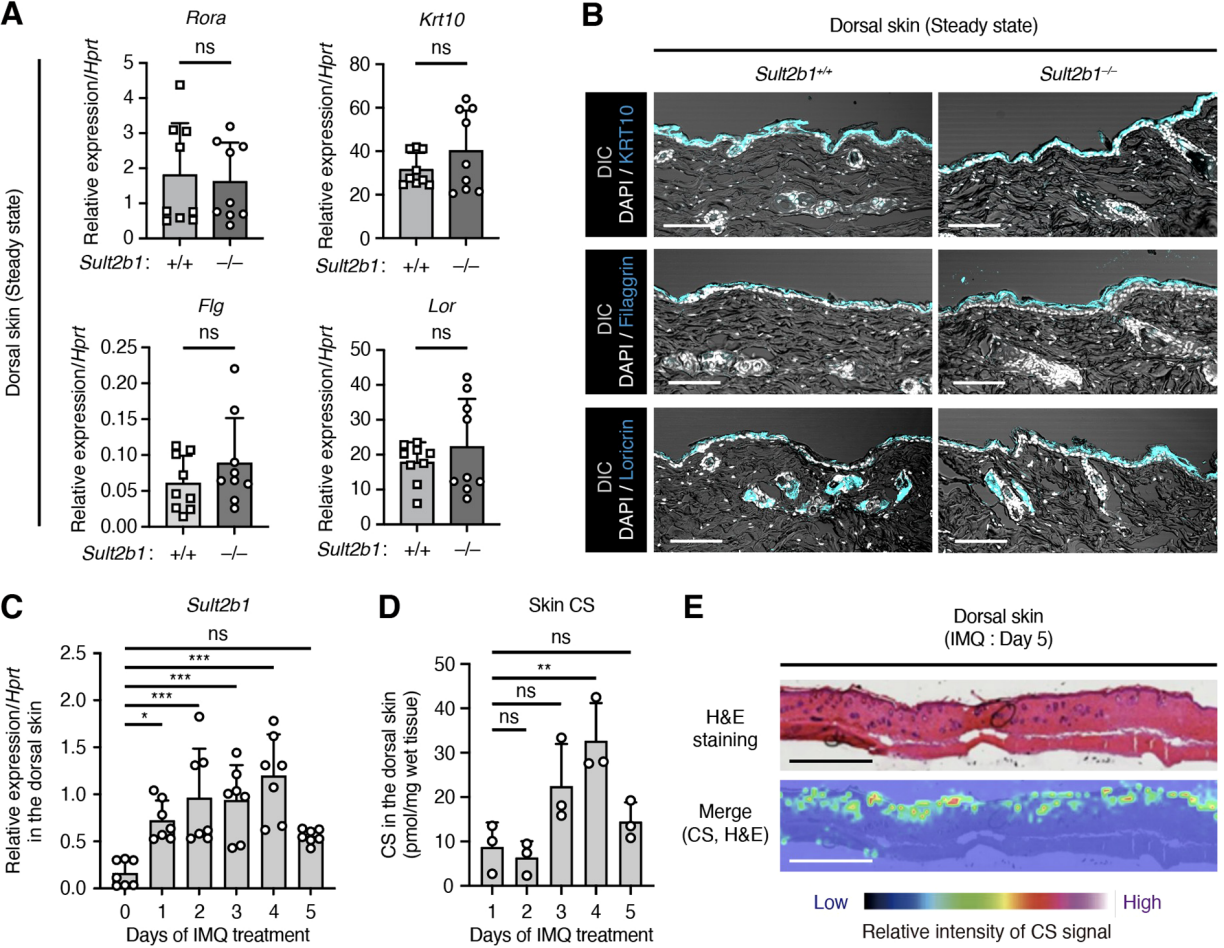
Imiquimod (IMQ)-induced skin inflammation upregulates the *Sult2b1* expression levels. **(A)** Real-time polymerase chain reaction (PCR) analysis of the barrier function-related gene expression levels in the dorsal skin of *Sult2b1^+/+^* and *Sult2b1^−/−^* mice at the steady state (n = 9 mice/group; two-tailed unpaired Student’s *t*-test). Target gene expression was normalized to that of hypoxanthine phosphoribosyltransferase (*Hprt*). **(B)** Representative IF staining images showing the KRT10, filaggrin, and loricrin (cyan) expression levels in the dorsal skin of *Sult2b1^+/+^* and *Sult2b1^−/−^* mice at the steady state counterstained with DAPI (nucleus; white). Scale bar, 100 μm. **(C, D)** Real-time PCR analysis of *Sult2b1* gene **(C)** and CS **(D)** levels in the dorsal skin of *Sult2b1^+/+^* mice treated with IMQ for five consecutive days. (n = 7 or 3 mice/group, respectively; one-way ANOVA, followed by Dunnett’s multiple-comparison test). *Sult2b1* gene expression was normalized to *Hprt* expression. **(E)** CS localization in the dorsal skin sections of *Sult2b1^+/+^* mice five days after IMQ application visualized via H&E staining and MS imaging. Color bar indicates the relative intensity of the CS signal (*m/z* 465). Scale bar, 1.0 mm. Data were obtained from three **(A, C)** and two **(B, D, E)** independent experiments, and graphs are represented as the mean ± SD. *P < 0.05, **P < 0.01, and ***P < 0.001; ns, not significant.

We further investigated the functional roles of SULT2B1 in the skin. A previous *in vitro* study using human epidermal KCs reported that high concentrations of exogenous CS enhance the skin barrier by inducing the retinoic acid receptor-related orphan receptor alpha (RORα) (28). Therefore, to determine the roles of endogenous CS *in vivo*, we compared the gene expression levels of transcription factor *Rora* (RORα) and *Krt10* (KRT10), *Flg*, and *Lor*, which are important for epidermal barrier formation, between *Sult2b1* ^+/+^ and *Sult2b1* ^−/−^ mice at the steady state. Notably, expression levels of these genes were comparable between the two groups (**Figure 2A**). Moreover, IF staining for KRT10, FLG, and LOR showed no difference in the localization pattern of protein expression between *Sult2b1* ^+/+^ and *Sult2b1* ^−/−^ mice (**Figure 2B**). These findings suggest that SULT2B1 and CS are dispensable for skin barrier development under normal conditions.

*SULT2B1* expression levels are higher in the skin of patients with psoriasis than in that of healthy subjects (29, 30). To determine whether *Sult2b1* expression and CS production are altered under psoriatic inflammatory conditions, we established an IMQ (toll-like receptor 7 agonist)-induced skin inflammation model. Although IMQ is a potent immune activator used to treat actinic keratosis, psoriasis-like skin flares are often induced in predisposed humans as a side effect (31). Daily topical application of IMQ to the shaved dorsal skin of mice leads to human psoriasis-like dermatitis, characterized by erythema, skin thickening, scaling, and recruitment of various immune cells (18, 19). When IMQ cream was applied to the shaved dorsal skin of WT mice, *Sult2b1* mRNA levels increased the following day and peaked on day 4 (**Figure 2C**). CS production also significantly increased two days after this increase in gene expression (**Figure 2D**), and uniform distribution of CS in the epidermis five days after IMQ application was not different from that observed at the steady state (**Figures 1A**, **2E**). Collectively, these results suggest that some inflammatory signals are associated with *Sult2b1* gene expression and, in turn, induce CS production in the skin.

### 3.3 SULT2B1 inhibits the progression of psoriasis-like phenotypes in mice

**Figure 3:**
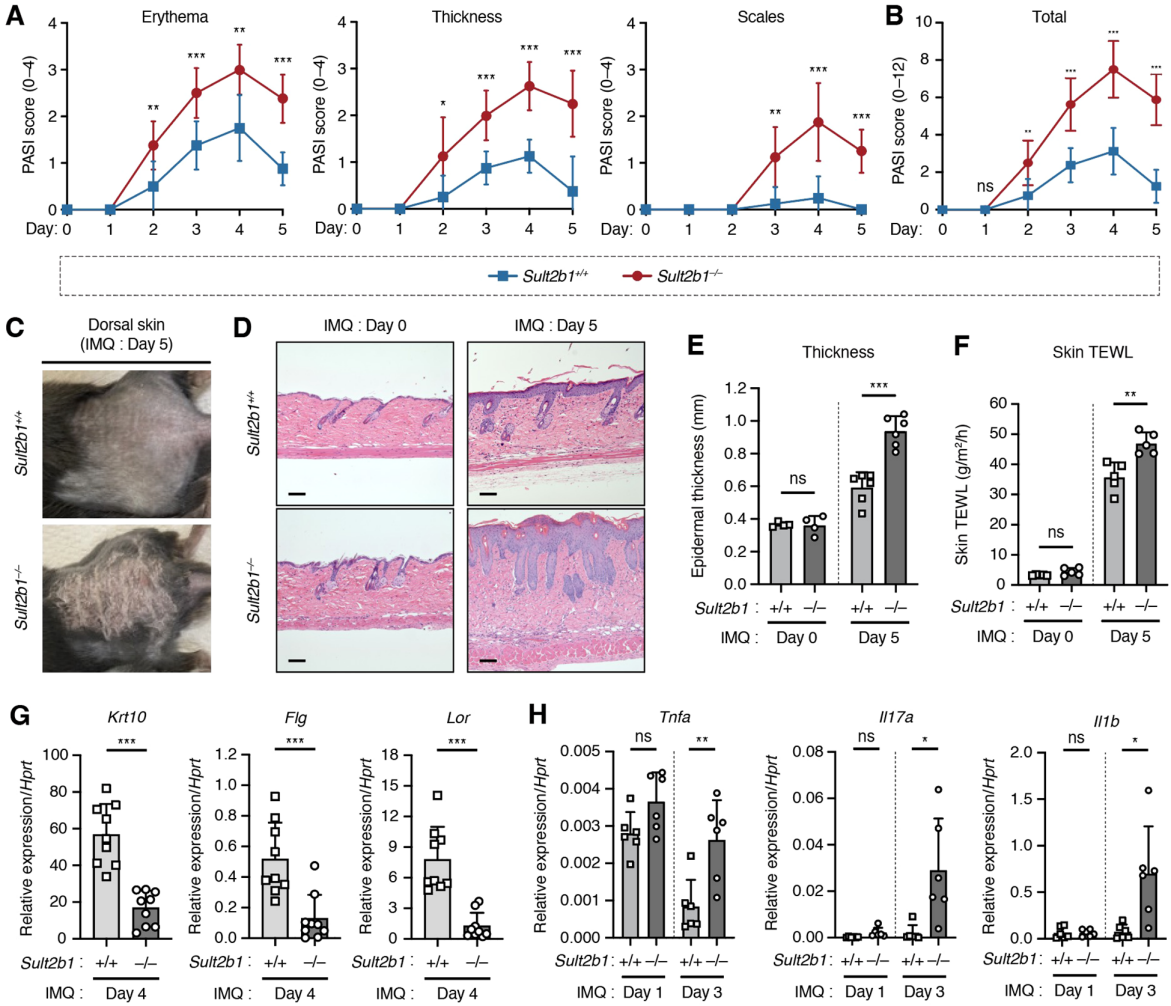
SULT2B1 inhibits the progression of psoriasis-like phenotypes in mice. **(A, B)** Daily psoriasis area and severity index (PASI) scores for each phenotype **(A)** and cumulative scores **(B)** of *Sult2b1^+/+^* and *Sult2b1^−/−^* mice in five consecutive days (n = 8 mice/group; two-tailed unpaired Student’s *t*-test). **(C)** Representative skin appearance of *Sult2b1^+/+^* (upper) and *Sult2b1^−/−^* (lower) mice five days after IMQ application. **(D, E)** Representative H&E staining **(D)** and quantification of epidermal thickness **(E)** of the dorsal skin sections of IMQ-treated *Sult2b1^+/+^* (upper) and *Sult2b1^−/−^* mice (lower) on days 0 (left) and 5 (right) (n = 6 mice/group; two-tailed unpaired Student’s *t*-test). Scale bar, 100 μm. **(F)** Trans epidermal water loss (TEWL) by the dorsal skin of IMQ-treated *Sult2b1^+/+^* and *Sult2b1^−/−^* mice on days 0 and 5 (n = 5 mice/group; two-tailed unpaired Student’s *t*-test). **(G, H)** Real-time PCR analysis of the barrier function-related **(G)** and pro-inflammatory gene **(H)** expression levels in the dorsal skin of *Sult2b1^+/+^* and *Sult2b1^−/−^* mice after IMQ application (n = 9 or 6 mice/group, respectively; two-tailed unpaired Student’s *t*-test). Target gene expression was normalized to *Hprt* expression. Data were obtained from three **(A–C, F–H)** and two **(D, E)** independent experiments, and graphs are represented as the mean ± SD. *P < 0.05, **P < 0.01, and ***P < 0.001; ns, not significant.

Functional significance of the elevated *Sult2b1* expression levels in the skin of WT mice under inflammatory conditions remains unknown. To evaluate whether and to what extent absence of SULT2B1 affects cutaneous inflammation, we applied IMQ cream to the shaved dorsal skin of mice for five consecutive days. Two days after IMQ application, *Sult2b1* ^−/−^ mice exhibited significant differences in the severity of erythema and skin thickening, and one day later they showed differences in the signs of scaling (**Figure 3A**). Notably, the overall symptoms were alleviated (**Figure 3B**), but the difference in skin phenotypes between *Sult2b1* ^+/+^ and *Sult2b1* ^−/−^ mice was still visible on day 5 (**Figures 3B, C**).

Hematoxylin and eosin staining of dorsal skin showed that the epidermis was considerably thicker in *Sult2b1* ^−/−^ mice than in *Sult2b1* ^+/+^ mice five days after IMQ application, but no difference in epidermal thickness was observed between *Sult2b1* ^+/+^ and *Sult2b1* ^−/−^ mice before IMQ application (**Figures 3D, E**). *Sult2b1* ^−/−^ mice also exhibited a higher TEWL rate than *Sult2b1* ^+/+^ mice after IMQ application (**Figure 3F**), indicating enhanced epidermal barrier disruption during cutaneous inflammation. Additionally, we evaluated the expression levels of barrier function-related genes *Krt10*, *Flg*, and *Lor* and found that their mRNA levels were markedly decreased in the dorsal skin of *Sult2b1* ^−/−^ mice (**Figure 3G**). Conversely, gene expression levels of the inflammatory cytokines associated with psoriasis, tumor necrosis factor (*Tnf*), interleukin-17a (*Il17a*), and *Il1b* (1–4), were significantly upregulated in *Sult2b1* ^−/−^ mice (**Figure 3H**). These results suggest that SULT2B1 inhibits the progression of psoriasis-like phenotypes, such as epidermal barrier disruption and cytokine hyperactivity.

### 3.4 Genetic deletion of *Sult2b1* exacerbates psoriatic dermatitis by increasing neutrophil infiltration

**Figure 4:**
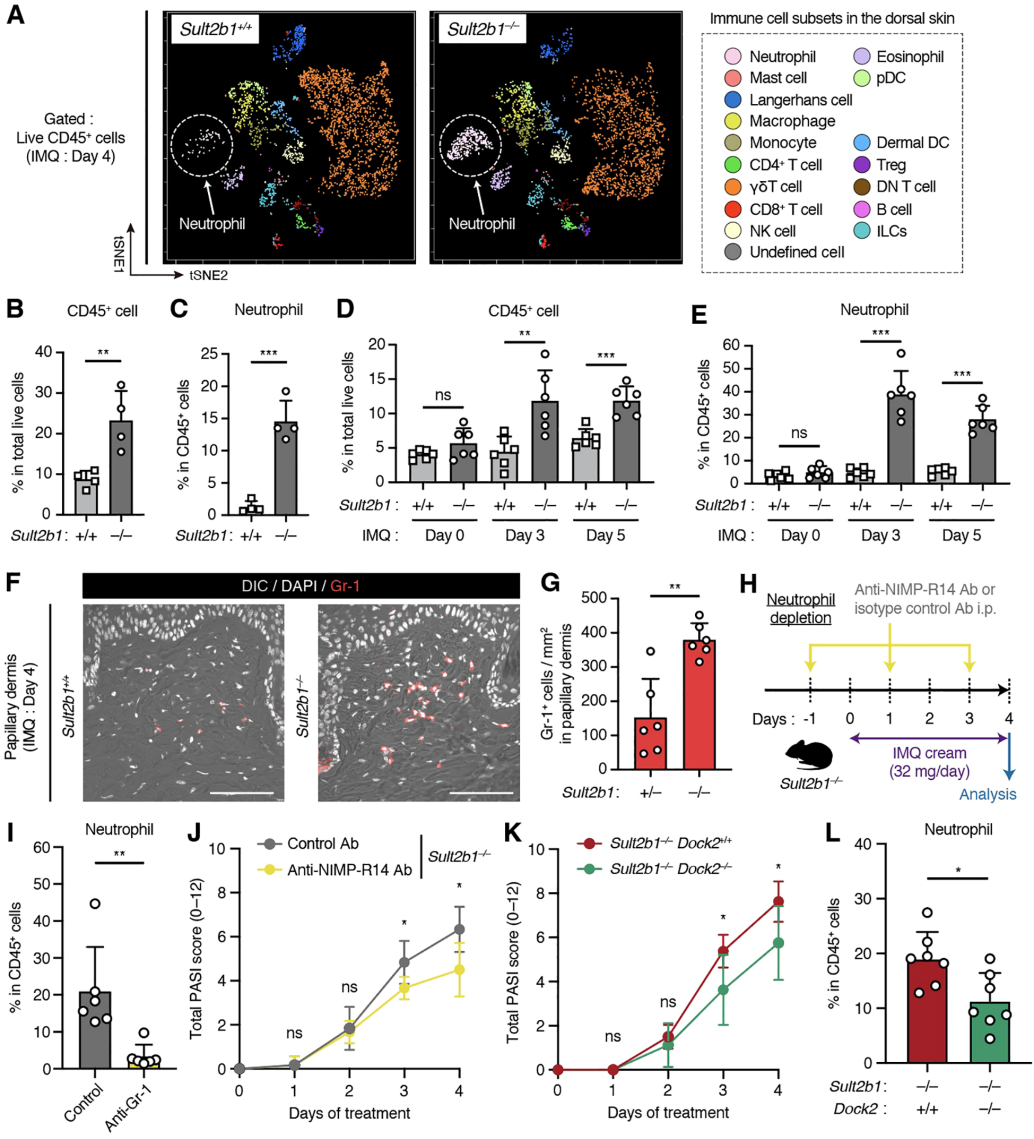
Genetic deletion of *Sult2b1* exacerbates psoriatic dermatitis by promoting neutrophil infiltration. **(A)** t-SNE visualization of the dermal immune cell subsets of *Sult2b1* ^+/+^ and *Sult2b1* ^−/−^ mice four days after IMQ application. **(B, C)** Percentages of CD45^+^ cells among the total live cells **(B)** and neutrophils among CD45^+^ cells **(C)** isolated from the dorsal skin of IMQ-treated *Sult2b1* ^+/+^ and *Sult2b1* ^−/−^ mice determined using cytometry by time-of-flight (CyTOF; each dot represents a pooled sample from four mice; n = 4/group; two-tailed unpaired Student’s *t*-test). **(D, E)** Percentages of CD45^+^ cells among total live cells **(D)** and neutrophils among CD45^+^ cells **(E)** isolated from the dorsal skin of IMQ-treated *Sult2b1* ^+/+^ and *Sult2b1* ^−/−^ mice determined via flow cytometry (n = 6 mice/group; two-tailed unpaired Student’s *t*-test). **(F, G)** Representative IF staining showing the Gr-1 (red) expression **(F)** levels and numbers of Gr-1^+^ DAPI^+^ cells in the papillary dermis **(G)** of *Sult2b1^+/+^* and *Sult2b1^−/−^* mice four days after IMQ application (n = 6 mice/group; two-tailed unpaired Student’s *t*-test). Scale bar, 100 μm. **(H)** Schematic illustration of the protocol used for neutrophil depletion. Indicated antibodies were intraperitoneally administered to *Sult2b1^−/−^* mice before and after IMQ application. **(I, J)** Percentage of neutrophils among CD45^+^ cells isolated from the dorsal skin tissues **(I)**, and total PASI scores **(J)** of IMQ-treated *Sult2b1^−/−^* mice with the indicated antibody treatments (n = 6 mice/group; two-tailed unpaired Student’s *t*-test). **(K, L)** Total PASI scores **(K)** and percentage of neutrophils among CD45^+^ cells isolated from the dorsal skin tissues **(L)** of IMQ-treated *Sult2b1^−/−^Dock2^+/+^* and *Sult2b1^−/−^Dock2^−/−^* mice (n = 7 mice/group; two-tailed unpaired Student’s *t*-test). Data were obtained from three **(A–L)** independent experiments, and graphs are represented as the mean ± SD. *P < 0.05, **P < 0.01, and ***P < 0.001; ns, not significant.

CS is synthesized by SULT2B1 and suppresses immune cell migration by inhibiting DOCK2-mediated Rac GEF activity (14). Therefore, we focused on whether the presence or absence of CS causes differences in the immune cell composition under cutaneous inflammatory conditions. To assess multiple immune cell subsets on the dorsal skin, we performed CyTOF analysis using metal-conjugated antibodies and the Helios mass cytometer. CyTOF is a high-throughput single-cell analysis combining two experimental platforms, flow cytometry and elemental MS, for the multidimensional characterization of cellular composition (32). After gating singlets and live CD45^+^ cells, we detected 17 immune cell subsets in the dorsal skin after IMQ application (**Figure 4A**, **Supplementary Figure S2**). Separation of these cell types was also confirmed via the differential expression of individual lineage markers and visualization via t-distributed stochastic neighbor embedding algorithm (**Supplementary Figure S3**). Percentage of CD45^+^ cells among total live cells isolated from skin was significantly higher in *Sult2b1* ^−/−^ mice than in *Sult2b1* ^+/+^ mice (**Figure 4B**). Among live CD45^+^ cells, neutrophils, CD8^+^ T cells, CD4^+^ T cells, Tregs, and B cells were more abundant in *Sult2b1* ^−/−^ mice (**Figure 4C**, **Supplementary Figure S4**), with a particularly marked difference in neutrophils (**Figures 4A, C**).

Next, we examined the changes in the percentage of neutrophils during IMQ-induced cutaneous inflammation using conventional flow cytometry. The percentages of CD45^+^ cells in total live cells and neutrophils in live CD45^+^ cells were unchanged between *Sult2b1* ^+/+^ and *Sult2b1* ^−/−^ mice at the steady state; however, these percentages were considerably higher in *Sult2b1* ^−/−^ mice than in *Sult2b1* ^+/+^ mice on days 3 and 5 (**Figures 4D, E**). Consistently, IF staining demonstrated that the number of Gr-1^+^ neutrophils infiltrating the papillary dermis just below the epidermis was significantly increased in *Sult2b1* ^−/−^ mice (**Figures 4F, G**). These results suggest that the infiltrating neutrophils contribute to the exacerbation of psoriatic dermatitis in *Sult2b1* ^−/−^ mice.

To verify this observation, we intraperitoneally administered the neutrophil depletion antibody, NIMP-R14, which is commonly used to remove neutrophils *in vivo* (26, 33), to *Sult2b1* ^−/−^ mice every other day (**Figure 4H**). Upon treatment with the NIMP-R14 antibody, the percentage of dermal neutrophils and the severity of dermatitis in *Sult2b1* ^−/−^ mice were decreased on day 4 after IMQ application (**Figures 4I, J**). Subsequently, we tested whether the genetic deletion of DOCK2, a CS target, alleviates severe inflammation in the skin of *Sult2b1* ^−/−^ mice. Notably, *Sult2b1* ^−/−^ *Dock2* ^−/−^ mice exhibited significant recovery of psoriasis-like phenotypes and neutrophil infiltration in the skin compared to *Sult2b1* ^−/−^ *Dock2* ^+/+^ mice (**Figures 4K, L**), suggesting that CS alleviates IMQ-induced psoriatic dermatitis by suppressing DOCK2 function.

### 3.5 Th1 cytokines promote *SULT2B1* mRNA expression in human KCs

**Figure 5:**
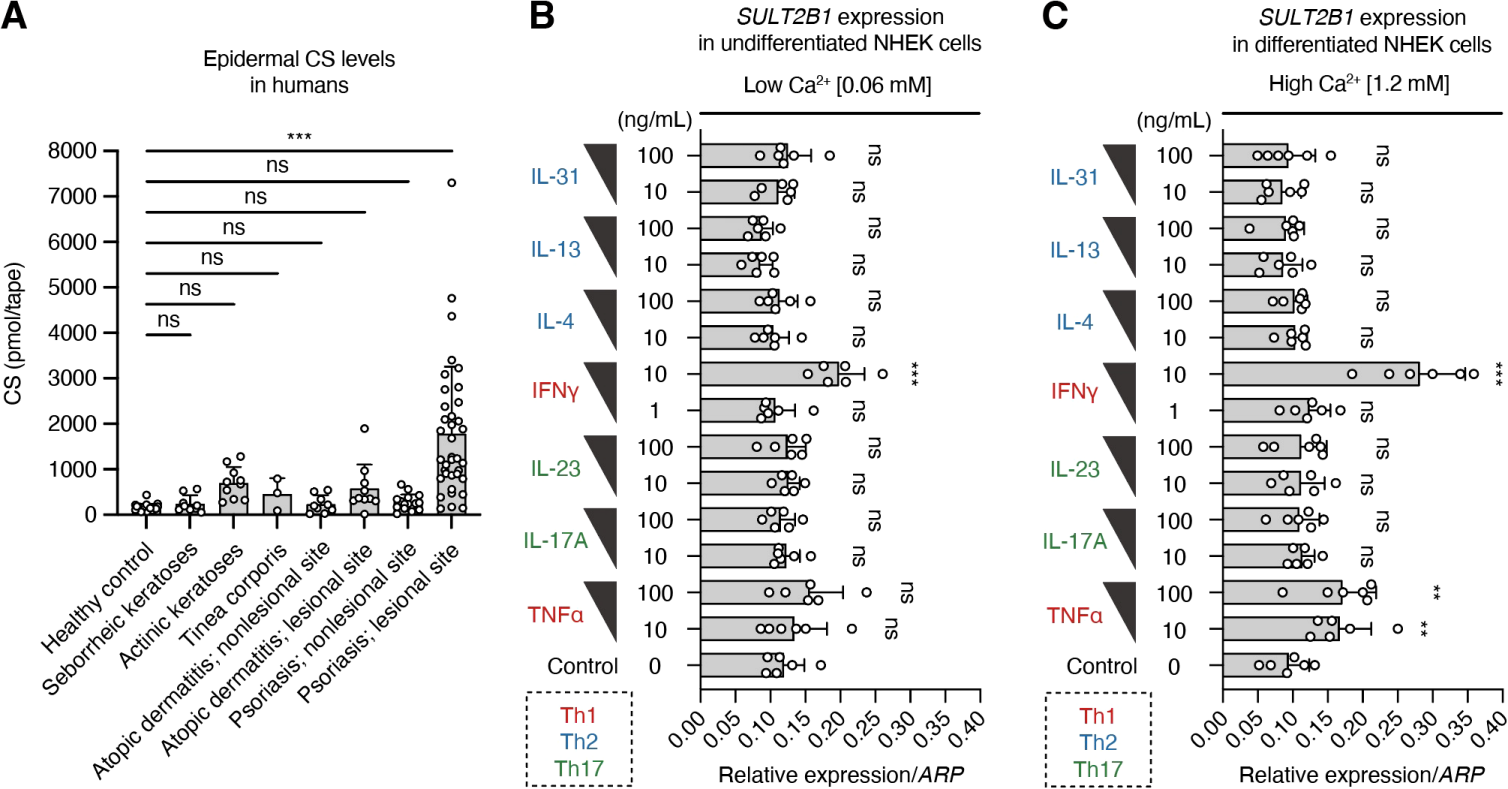
T-helper (Th)-1 cytokines promote *SULT2B1* mRNA expression in human keratinocytes. **(A)** Epidermal CS levels in healthy controls (n = 30) and patients with the indicated skin diseases [seborrheic keratoses (n = 9), actinic keratoses (n = 10), tinea corporis (n = 3), atopic dermatitis (non-lesional, n = 9; lesional, n = 10), and psoriasis (non-lesional: n = 16; lesional: n = 37)] quantified via supercritical fluid chromatography-tandem mass spectrometry (SFC-MS/MS; one-way ANOVA, followed by Dunnett’s multiple-comparison test). **(B, C)** Real-time PCR analysis of *SULT2B1* gene levels in undifferentiated **(B)** or differentiated **(C)** normal human epidermal keratinocytes (NHEKs) treated with two concentrations of the indicated cytokines (n = 6/group; one-way ANOVA, followed by Dunnett’s multiple-comparison test). Data were obtained from six **(B, C)** independent experiments, and graphs are represented as the mean ± SD. **P < 0.01 and ***P < 0.001; ns, not significant.

A previous study on human abdominal skin detected CS in the epidermal region just below the stratum corneum, which is composed of dead cells in the outermost skin layer (34). However, little is known regarding the levels of CS in various skin diseases involving inflammation. Therefore, we reanalyzed and compared the epidermal CS levels among common skin diseases, such as seborrheic keratoses, actinic keratoses, tinea corporis, atopic dermatitis, and psoriasis, using the previously published lipid-profiling data (23) of human tape stripping samples. Epidermal CS levels were significantly higher in patients with psoriasis than in the healthy controls (**Figure 5A**). Moreover, CS was more abundant at lesional sites than at anatomically matched non-lesional sites in patients with psoriasis. These results suggest increased CS production as a characteristic change in psoriatic lesions.

Some pro-inflammatory cytokines secreted mainly by Th cells possibly regulate CS production by promoting *SULT2B1* gene expression in psoriatic skin, where interactions between Th cells and KCs have been reported (1–4). To verify this, we cultured the primary NHEKs isolated from adult skin and treated them with representative Th1, Th2, and Th17 cytokines. Under normal low Ca^2+^ medium conditions, interferon (IFN)-γ upregulated the *SULT2B1* mRNA levels in undifferentiated NHEKs (**Figure 5B**). When cultured in the high Ca^2+^ medium for 72 h to induce keratinization (terminal differentiation), NHEKs promoted the *SULT2B1* mRNA levels after treatment with TNFα and IFNγ (**Figure 5C**). However, addition of IL-4, IL-13, IL-17A, IL-23, and IL-31 to the culture medium did not cause any differences in gene expression levels compared to those in the controls. These findings suggest that Th1 cytokines are important for *SULT2B1* upregulation in psoriasis.

## 4 Discussion

In this study, we found that SULT2B1 expression and CS production in the epidermis may function as an endogenous regulatory axis that helps to limit excessive immune responses under cutaneous inflammation. Using a murine model of psoriasis-like dermatitis induced by topical IMQ, we found that the loss of SULT2B1 was associated with exacerbated skin inflammation and infiltration of neutrophils, which are central to psoriatic pathology. Mechanistically, these phenotypes were alleviated by neutrophil depletion and genetic deletion of DOCK2, a Rac GEF and CS target. These findings support the idea that SULT2B1 may play homeostatic and anti-inflammatory roles in EIME by modulating immune cell dynamics.

CS directly binds to DOCK2 and suppresses its activity, thereby limiting the Rac-dependent migration and activation of immune cells (14). Our current data extend this concept by revealing its physiological relevance to the skin, a barrier organ constantly exposed to environmental stimuli and prone to inflammatory diseases. Notably, we found that epidermal KCs were the major producers of SULT2B1 and that *Sult2b1* mRNA levels were significantly upregulated in response to IMQ treatment (**Figures 1**, **2**). Despite a previous report indicating that CS increases profilaggrin expression via induction of the transcription factor RORα and is involved in epidermal differentiation in human NHEKs (28), our data using *Sult2b1* ^−/−^ mice suggest that SULT2B1 and CS are not essential for skin barrier formation under at least homeostatic conditions (**Figures 2A, B, E, F**). Absence of SULT2B1 exacerbated psoriatic inflammation in mice, as evidenced by the enhanced epidermal thickening and TEWL, downregulated skin barrier gene (*Krt10*, *Flg*, and *Lor*) levels, and upregulated inflammatory cytokine (*Il17a*, *Tnf*, and *Il1b*) levels (**Figure 3**). These observations suggest that the inflammatory response mechanisms are involved in CS generation and may regulate immune overactivation.

*Sult2b1* ^−/−^ mice exhibited increased neutrophil recruitment, highlighting the central role of innate immunity in mediating the pathological consequences of CS deficiency (**Figure 4**). Neutrophils are abundant in psoriatic lesions (35) and contribute to disease propagation by releasing pro-inflammatory mediators and neutrophil extracellular traps (36). Indeed, neutrophil depletion ameliorated these symptoms in *Sult2b1* ^−/−^ mice. In addition, our CyTOF data also demonstrated enhanced infiltration of CD4^+^ T, CD8^+^ T, and B cells in *Sult2b1* ^−/−^ mice compared to that in *Sult2b1* ^+/+^ mice (**Supplementary Figure S4**). Similarly, a clinical study of patients with a loss-of-function mutation in *SULT2B1* revealed the loss of epidermal CS and numerous perivascular lymphocytic infiltrates in the epidermis (37), suggesting the key role of the SULT2B1–CS–DOCK2 axis in limiting neutrophilic and lymphocytic inflammation in the skin. In psoriatic skin, immune subsets including Th17 cells, γδ T cells, and dendritic cells are increased or activated along with neutrophils (38–40). In this study, CyTOF profiling without intracellular staining could not identify Th17 cells but revealed that the proportion of CD4^+^ T cells increased in *Sult2b1* ^−/−^ mice (**Supplementary Figure S4**). Considering the significantly elevated *Il17a* gene expression levels in the skin of *Sult2b1* ^−/−^ mice after IMQ application (**Figure 3H**), SULT2B1 may suppress Th17 cell infiltration and/or activation during psoriatic skin inflammation. Furthermore, the proportions of γδ T cells, Langerhans cells, dermal DCs, and pDCs were unaffected by SULT2B1 (**Supplementary Figure S4**). However, the relationship between SULT2B1 and the activation and cytokine production of these cell types remains unclear and warrants further investigation.

EIME, composed of both immune and non-immune cells, acts as a frontline defense against environmental insults and pathogens (1–3). Inflammatory cytokines modulate KC activation and drive complex immune circuits implicated in various diseases, such as psoriasis. In this study, epidermal CS levels were specifically elevated in the lesional skin of patients with psoriasis, but not in the skin of patients with other common dermatoses (**Figure 5**), suggesting CS as a predominant lipid biomarker for psoriasis. A previous study also showed elevated CS levels in the skin of patients with psoriasis (41). Here, IFNγ and TNFα, the key Th1 cytokines implicated in psoriasis pathogenesis, induced *SULT2B1* expression in human epidermal KCs, whereas Th2 and Th17 cytokines did not alter *SULT2B1* expression. These results provide a novel mechanistic explanation for the increased *SULT2B1* mRNA and CS levels in psoriatic lesions and highlight the crucial role of the Th1-driven immune–epithelial crosstalk in maintaining EIME homeostasis. To our knowledge, this is the first report demonstrating an association between specific inflammatory cytokines and *SULT2B1* expression, necessitating further analysis of the detailed expression regulatory mechanisms in both *in vitro* and *in vivo* settings.

This study has some limitations. First, although SULT2B1 expression correlates with epithelial and immune responses, the causal link remains underdeveloped. We previously showed that CS inhibits the DOCK2-Rac pathway, suppressing neutrophil migration and reactive oxygen species (ROS) production (14, 20). In *Sult2b1* ^−/−^ *Dock2* ^−/−^ mice, skin inflammation was improved compared to *Sult2b1* ^−/−^ *Dock2* ^+/+^ mice (**Figure 4**). However, if only the SULT2B1-CS-DOCK2 axis is affected in psoriasis-like dermatitis, psoriatic phenotypes in *Sult2b1* ^−/−^ *Dock2* ^−/−^ mice would be expected to decrease to WT levels (**Figures 3B**, **4K**). Therefore, SULT2B1 likely provides not only immunosuppression but also other skin-protective effects. In KC biology, SULT2B1 and CS have been reported to regulate epidermal differentiation and barrier function (16, 28, 42, 43). Together, these findings suggest that CS could act on both KCs and immune cells to contribute to EIME homeostasis. Second, while this study revealed that epidermal KCs are the primary cells expressing *Sult2b1* in the skin, it lacks functional assays to prove whether SULT2B1 produced in KCs directly contributes to the severity of psoriatic skin inflammation, leaving it limited to suggesting a correlation. Therefore, future studies could further validate the KC-intrinsic immune regulatory mechanism mediated by SULT2B1 through perturbation experiments, such as using KC-specific *Sult2b1* knockout or overexpression mice, or introducing *Sult2b1* mRNA/siRNA into skin KCs via drug delivery systems.

To date, nuclear receptors such as liver X receptors (LXRs) and peroxisome proliferator-activated receptors (PPARs) have been identified as regulators enhancing SULT2B1 expression in KCs (44–46). Therefore, CS production may be influenced by genetic and environmental factors that increase or decrease the expression of these lipid metabolism regulators. In this study, our findings suggest that SULT2B1 contributes to EIME homeostasis, at least partially by limiting neutrophil infiltration and inflammatory cytokine expression in the skin. Within the broader landscape of psoriasis biology, SULT2B1 is positioned as a key metabolic enzyme forming a complex epithelial-immune network. In summary, the significance of this study lies in demonstrating that SULT2B1 and its product, CS, not only participate in epidermal differentiation and barrier formation but also act as suppressive modulators of inflammation and immune cell behaviors in the psoriatic environment. These findings provide novel insights into the pathogenesis of psoriasis, facilitating the development of new therapeutic approaches for this disease.

## Data Availability

Publicly available datasets were analyzed in this study. This data can be found here: https://tabula-muris.sf.czbiohub.org.
