# Supplementary material for: Sulfotransferase SULT2B1 contributes to the epithelial–immune microenvironment homeostasis in imiquimod-induced psoriatic dermatitis
Source: Front Immunol. 2025 Oct 17;16:1632426. doi: 10.3389/fimmu.2025.1632426 (PMC12575227; doi:10.3389/fimmu.2025.1632426)
Supplement: Supplementary file 1 [file DataSheet1.pdf]

Supplementary Material

1 Supplementary Figures and Tables

1.1 Supplementary Figures

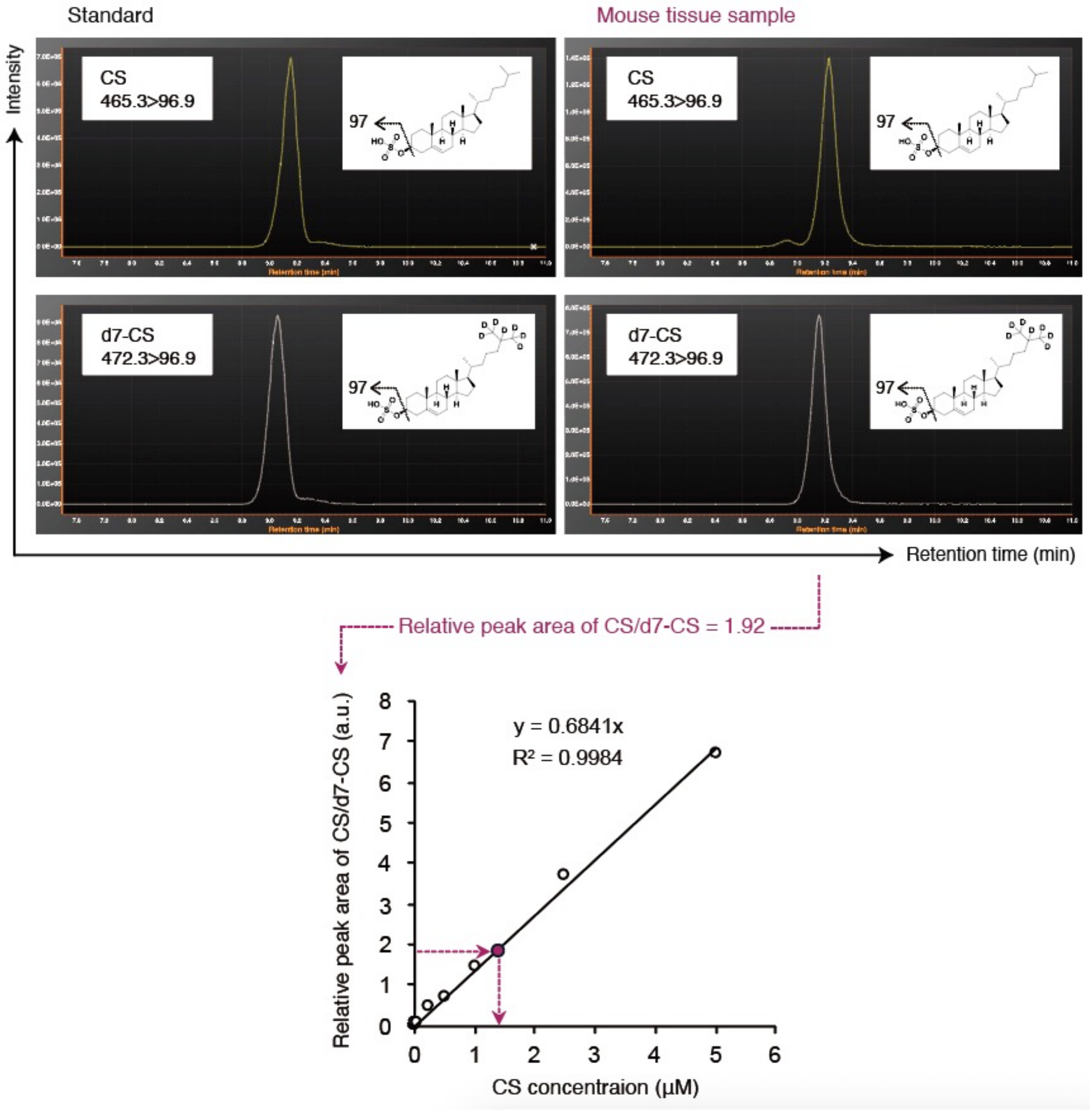

Supplementary Figure 1. Representative chromatograms and calibration curves of CS, analyzed using LC–MS/MS. The signals for CS and d7-CS were monitored as MRM transitions at *m/z* 465.3 > 96.9 and 472.3 > 96.9, respectively. The area under the extracted-ion chromatogram peak of CS was divided

by that of the internal standard (d7-CS), and the area ratio was plotted against the amount of the species spiked. The equation for the straight line was used to calculate the slope, while the correlation coefficient (R^2) was calculated from regression analysis. A linear relationship was found between analyte concentration and relative peak areas. The resulting calibration curve, data points, and R^2 value are shown (n = 3 technical replicates).

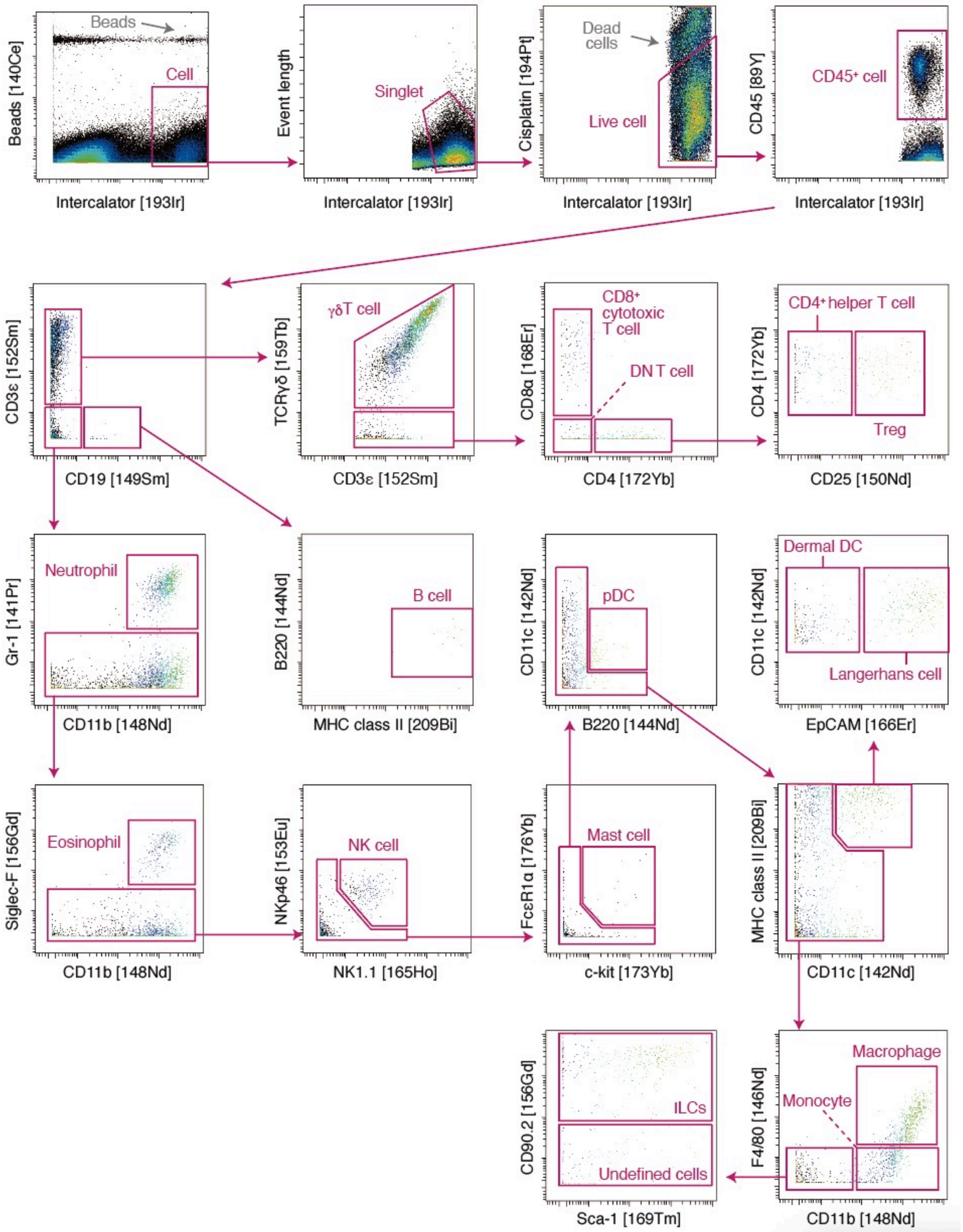

Supplementary Figure 2. Gating strategy for highly multiparametric analysis by CyTOF. After gating single viable CD45⁺ immune cells, each immune cell type was identified using the following markers: B cell (CD3ε⁻ CD19⁺ B220⁺ MHCII⁺), γδT cell (CD3ε⁺ CD19⁻ TCRγδ⁺), helper T cell (CD3ε⁺ CD19⁻ TCRγδ⁻ CD4⁺ CD8α⁻ CD25⁻), regulatory T cell (Treg; CD3ε⁺ CD19⁻ TCRγδ⁻ CD4⁺ CD8α⁻ CD25⁺), cytotoxic T cell (CD3ε⁺ CD19⁻ TCRγδ⁻ CD4⁻ CD8α⁺), double-negative T cell (DN T cell; CD3ε⁺

$CD19^{-}$ $TCR\gamma\delta^{-}$ $CD4^{-}$ $CD8\alpha^{-}$), neutrophil ($CD3\varepsilon^{-}$ $CD19^{-}$ $Gr\text{-}1^{+}$ $CD11b^{+}$), eosinophil ($CD3\varepsilon^{-}$ $CD19^{-}$ $Gr\text{-}1^{-}$ $CD11b^{+}$ $Siglec\text{-}F^{+}$), natural killer cell (NK cell; $CD3\varepsilon^{-}$ $CD19^{-}$ $Gr\text{-}1^{-}$ $Siglec\text{-}F^{-}$ $NK1.1^{+}$ $NKp46^{+}$), mast cell ($CD3\varepsilon^{-}$ $CD19^{-}$ $Gr\text{-}1^{-}$ $Siglec\text{-}F^{-}$ $c\text{-}kit^{+}$ $Fc\varepsilon RI^{+}$), plasmacytoid dendritic cell (pDC; $CD3\varepsilon^{-}$ $CD19^{-}$ $Gr\text{-}1^{-}$ $Siglec\text{-}F^{-}$ $CD11c^{int}$ $B220^{+}$), dermal DC ($CD3\varepsilon^{-}$ $CD19^{-}$ $Gr\text{-}1^{-}$ $Siglec\text{-}F^{-}$ $CD11c^{+}$ $MHCII^{+}$ $EpCAM^{-}$), Langerhans cell ($CD3\varepsilon^{-}$ $CD19^{-}$ $Gr\text{-}1^{-}$ $Siglec\text{-}F^{-}$ $CD11c^{+}$ $MHCII^{+}$ $EpCAM^{+}$), monocyte ($CD3\varepsilon^{-}$ $CD19^{-}$ $Gr\text{-}1^{-}$ $Siglec\text{-}F^{-}$ $CD11b^{+}$ $F4/80^{-}$), macrophage ($CD3\varepsilon^{-}$ $CD19^{-}$ $Gr\text{-}1^{-}$ $Siglec\text{-}F^{-}$ $CD11b^{+}$ $F4/80^{+}$), ILCs ($CD3\varepsilon^{-}$ $CD19^{-}$ $Gr\text{-}1^{-}$ $Siglec\text{-}F^{-}$ $CD11b^{-}$ $F4/80^{-}$ $CD90.2^{+}$).

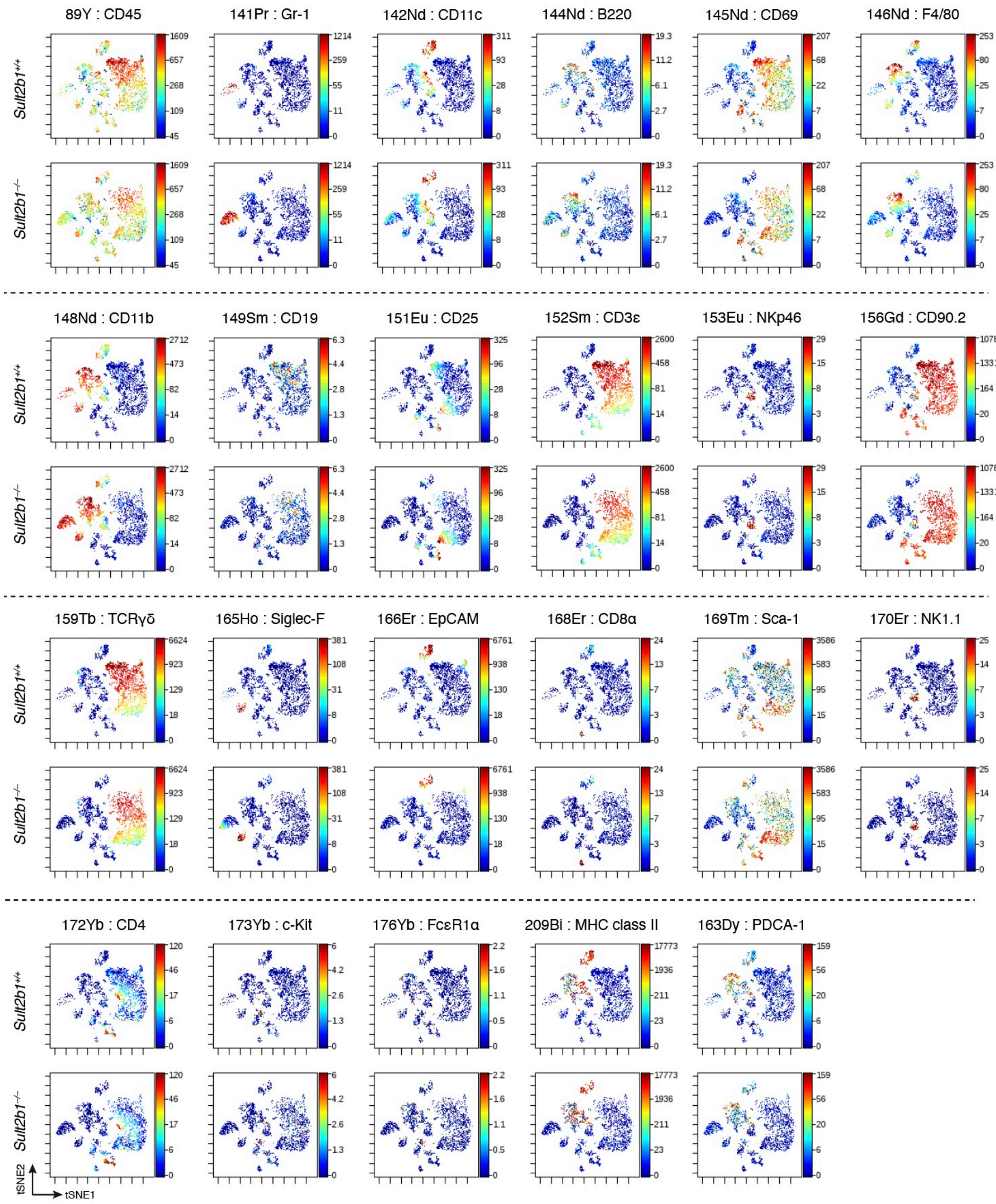

Supplementary Figure 3. t-SNE plots after data concatenation (n = 4) overlaid with the expression heatmaps of individual markers. Red and blue indicate high and low expression, respectively. IMQ-treated *Sult2b1*$^{+/+}$ and *Sult2b1*$^{-/-}$ mice on day 4 were shown in the upper and lower panels, respectively.

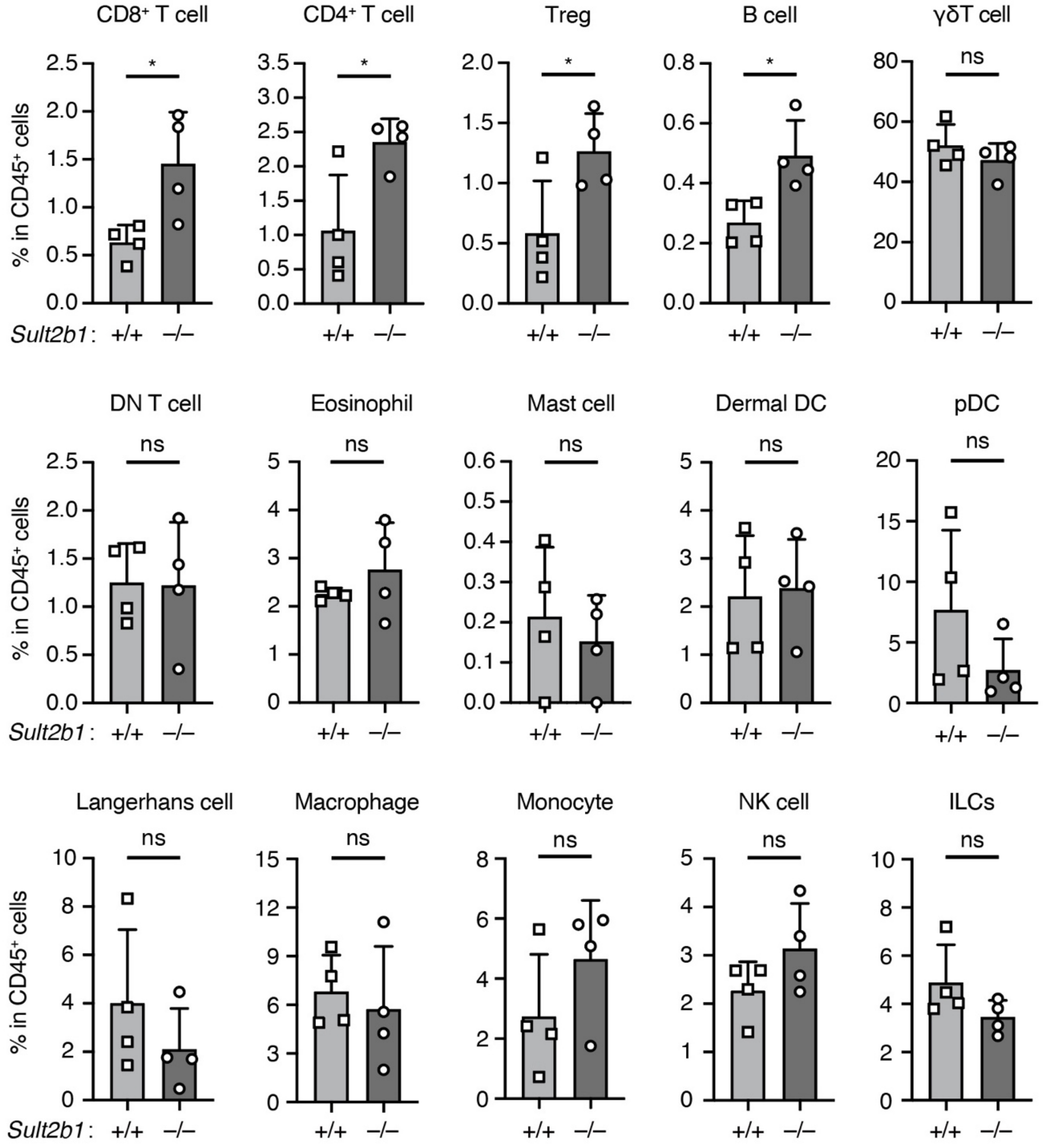

Supplementary Figure 4. Percentages of skin tissue-infiltrating immune cell subsets among $CD45^+$ cells 4 days after IMQ application, analyzed by CyTOF (n = 4 mice per group; two-tailed unpaired Student's *t*-test). Data were obtained from four independent experiments, and graphs are shown as the mean ± SD. *$P < 0.05$; ns, not significant.

Supplementary Table 1. List of real-time PCR primer sequences.

Gene	Forward Sequence (5'− 3')	Reverse Sequence (5'− 3')
**Mouse**		
Il17a	TAACTCCCTTGGCGCAAAAG	TCTTCATTGCGGTGGAGAGT
Il1b	GAAGAAGAGCCCATCCTC	GTTCATCTCGGAGCCTGTAG
Tnfa	TCGTAGCAAACCACCAAGTG	TTTGAGATCCATGCCGTTGG
Sult2b1	GTTGGACATGGTATTGGCCT	TGCAACGCATCTGTGAGTTC
Krt10	AGAAGCATGGCAACTCAAGC	TGGCATTGTCAGTTGTCAGG
Flg	GACATCGCTGAAGTCTTCATGC	TTTGCCAGCTTTAGCACCAG
Lor	#qMmuCID0008842 (Bio-Rad)	#qMmuCID0008842 (Bio-Rad)
Rora	ATTGTCGGCTGCAGAAATGC	CTTGGACACCGACCAAACTTG
Hprt	CTGGTGAAAAGGACCTCTCG	TGAAGTACTCATTATAGTCAAGGGCA
**Human**		
SULT2B1b	ATGACATCTCGGAAATCAGCCA	GCACATCTTGGGTGTTCTCCG
RPLP0	GCTTCCTGGAGGGTGTCC	GGACTCGTTTGTACCCGTTG

Supplementary Table 2. CyTOF antibody panel used to examine dermal immune cell subsets.

No.	Metal conjugate	Target	Amount (mL)	Clone	Product ID (Fluidigm)
1	89Y	CD45	1	30-F11	3089005
2	141Pr	Ly-6G/C (Gr-1)	1	RB6-8C5	3141005
3	142Nd	CD11c	1	N418	3142003
4	144Nd	CD45R (B220)	1	RA3-6B2	3144011
5	145Nd	CD69	1	H1.2F3	3145005
6	146Nd	F4/80	1	BM8	3146008
7	148Nd	CD11b (Mac-1)	1	M1/70	3148003
8	149Sm	CD19	1	6D5	3149002
9	151Eu	CD25 (IL-2R)	1	3C7	3151007
10	152Sm	CD3e	1	145-2C11	3152004
11	153Eu	CD335 (NKp46)	1	29A1.4	3153006
12	156Gd	CD90.2/Thy1.2	1	30-H12	3156006
13	159Tb	TCRgd	1	GL3	3159012
14	163Dy	APC	1	APC003	3163001
15	165Ho	PE	1	PE001	3165015
16	168Er	CD8a	1	53-6.7	3168003
17	169Tm	Ly-6A/E (Sca-1)	1	D7	3169015
18	170Er	CD161 (NK1.1)	1	PK136	3170002
19	172Yb	CD4	1	RM4-5	3172003
20	173Yb	CD117 (c-Kit)	3	2B8	3173004
21	176Yb	FceRIa	1	1-Mar	3176006
22	209Bi	I-A/I-E (MHC class II)	1	M5/114.15.2	3209006
	**Fluorophore conjugate**	**Target**	**Amount (mL)**	**Clone**	**Product ID**
1	APC	BST2 (CD317)	1	927	127015
2	PE	Siglec-F	1	E50-2440	552126

Supplementary Table 3. List of human cytokines used for *in vitro* experiments.

**Cytokine**	**Product ID**	**Company**
HumanKine,<br>recombinant human TNF alpha protein	HZ-1014	Proteintech<br>(Rosemont, IL, USA)
HumanKine,<br>recombinant human IL-17 (IL-17A) protein	HZ-1113	Proteintech<br>(Rosemont, IL, USA)
HumanKine,<br>recombinant human IL-23 protein	HZ-1254	Proteintech<br>(Rosemont, IL, USA)
Human IL-4,<br>Animal-Free Recombinant Protein	AF-200-04	PeproTech<br>(Cranbury, NJ, USA)
Human IL-13,<br>Animal-Free Recombinant Protein	AF-200-13	PeproTech<br>(Cranbury, NJ, USA)
Human IFN-gamma,<br>Animal-Free Recombinant Protein	AF-300-02	PeproTech<br>(Cranbury, NJ, USA)
Recombinant Human Interleukin-31	cyt-625	ProSpec<br>(Rehovot, Israel)
